# Genetic Characterization of Chikungunya Virus Among Febrile Dengue Fever–Like Patients in Xishuangbanna, Southwestern Part of China

**DOI:** 10.3389/fcimb.2022.914289

**Published:** 2022-06-27

**Authors:** Meng Zou, Chunyan Su, Tingting Li, Jing Zhang, Daiying Li, Ning Luan, Dehong Ma, Jiansheng Liu, Qiangming Sun, Xiaozhong Peng, Hongqi Liu

**Affiliations:** ^1^ Institute of Medical Biology, Chinese Academy of Medical Science and Peking Union Medical School, Kunming, China; ^2^ Joint Laboratory for Prevention and Control of Cross-border Transmission Disease, People’s Hospital of Xishuangbanna Dai Autonomous Prefecture, Jinghong, China; ^3^ State Key Laboratory of Medical Molecular Biology, Department of Molecular Biology and Biochemistry, Institute of Basic Medical Sciences, Medical Primate Research Center, Neuroscience Center, Chinese Academy of Medical Sciences, School of Basic Medicine, Peking Union Medical College, Beijing, China

**Keywords:** Chikungunya virus, dengue virus, co-infection, adaptive mutation, vector-borne diseases

## Abstract

Co-infection of chikungunya virus (CHIKV) has been recently reported during dengue fever epidemics. However, the infection of CHIKV is often neglected due to its misdiagnosis as dengue virus (DENV) infection. In the summer of 2019 when dengue fever was epidemic, we collected 697 serum samples from febrile dengue fever–like patients in Xishuangbanna, southwestern part of China. DENV RNA was detectable in 99.42% of these patients. Notably, 88 patients (12.62%) showed the presence of CHIKV RNA, among which 86 patients were co-infected with DENV and CHIKV. We sequenced and analyzed the full genome of CHIKV virus in four out of 88 samples (two CHIKV infected and two co-infected). The results suggested that the four strains were all Asian genotype and had the highest homology (99.4%) with the SZ1239 strain (accession number MG664851) isolated in 2012 and possibly introduced from Indonesia. Further comparison with the conserved sequences in the whole genome of 47 strains of CHIKV showed that there were 13 and 15 amino acid mutants in structural proteins and non-structural proteins, respectively. The previously reported adaptive mutations of E2-W64R, E2-I211T, E2-K233E, E1-A98T, and E1-K211E occurred in the four strains of this study. In conclusion, this study reports a co-infection of CHIKV during the DENV epidemic in the city Xishuangbanna, 2019. Molecular epidemiology revealed that CHIKV identified in this study was indigenous and belongs to Asian lineage with lineage-specific mutations and some reported adaptive mutations, which is distinct from the recently reported CHIKV (East/Central/South African) in Ruili, the city next to Xishuangbanna.

## Introduction

Arboviral diseases, caused by arthropod-borne viruses, pose a global threat to global public health, which is recently called for priorities for research and development (Wilder-Smith et al., 2017). Arthropod vectors, essential for arbovirus transmission cycle, are mostly confined to specific ecological niches. Therefore, arboviral diseases are usually recognized as natural focal diseases. However, because of global changes of climate and socioeconomics and increase in international travel, viruses and vectors are spreading and emerging in new geographical areas where residents have no antibody or effective treatment against these pathogens. In addition, adaptive mutation of virus leads to transcontinental transmission and outbreak of disease in some unexpected regions (Tsetsarkin et al., 2007).

Dengue and chikungunya diseases are mostly concerned arboviral diseases due to their morbidity, mortality, and socioeconomic burden. Dengue disease is caused by dengue virus (DENV), which consists of four serotypes (I, II, III, and IV), belonging to genus *Flavivirus* of the *Flaviviridae* family (Wen et al., 2018). Chikungunya disease is resulted due to infection of chikungunya virus (CHIKV) that is a member of the genus *Alphavirus*, *Togavirida*e family (Vasconcellos et al., 2019). Although DENV and CHIKV belong to two different viral families, they are both transmitted by the common vector *Aedes mosquito*. In fact, cocirculation of DENV and CHIKV has been documented in many locations (Raza et al., 2021). However, higher incidence of DENV and similar clinical manifestations contribute to neglect of CHIKV detection in patients with DENV or even misdiagnosis of CHIKV as DENV, which may lead to serious consequences. For this purpose, multiplex RT-PCR (Reverse Transcription-Polymerase Chain Reaction) has been developed to detect DENV, CHIKV, and ZIKV (Zika virus) in single tube/well, by which co-infections of DENV, CHIKV, and/or ZIKV are actually found (Sánchez-Arcila et al., 2020). Notably, during dengue fever outbreak in 2019, there are two reports of CHIKV infection in Ruili city, Yunnan Province China, where CHIKV belongs to East/Central/South African (ECSA) lineage and are imported from Southeast Asian countries (Liu et al., 2021; Yin et al., 2021). Therefore, it is important to carry out surveillance of CHIKV. Here, we report a co-infection of CHIKV during dengue fever outbreak in Xishuangbanna and describe the viral origin of Asian lineage.

## Martials and Methods

### Ethics Statement

This study was approved by ethics committees of Xishuangbanna Dai Autonomous Prefecture People’s Hospital, Yunnan, China. Informed consent was written and obtained from each participant.

### Sample Collection and Laboratory Diagnosis

From September to November of 2019, we collected 697 serum samples from febrile dengue fever–like outpatients in Xishuangbanna Dai Autonomous Prefecture People’s Hospital, Yunnan, China. Total RNA was extracted and purified from serum samples according to the manufacturer’s instructions. Briefly, the serum samples were separated from the collected blood, followed by viral RNA extraction. Viral RNA was extracted from 200 µl of CHIKV-infected serum using the AxyPrep Body Fluid Viral DNA/RNA Miniprep Kit (Axygen, Inc., USA). The RNA was eluted in 50 µl of nuclease-free water and stored at −80°C. Multiplex one-step RT-PCR was performed to amplify gene fragments *via* specific primers for DENV (I–IV), CHIKV, and ZIKV (Calvo et al., 2016). The amplified products were visualized *via* agarose electrophoresis. The DNA band with the expected size was cut and purified for Sanger sequencing. Sequences of PCR products were aligned with published viral sequences to confirm the specific pathogens.

### Genome Sequencing

Viral RNA was extracted with an AxyPrep Body Fluid Viral DNA/RNA Miniprep Kit (Axygen, Inc., USA) according to the manufacturer’s instructions. Viral RNA was reversely transcribed into cDNA using the PrimeScript™ II First Strand cDNA Synthesis Kit (Takara Bio, Shiga, Japan). The cDNA was amplified by PCR *via* 12 pairs of overlapping primers (**Supplementary Table 2**). PCR was performed with the following protocol (50-µl volume): denaturation at 94°C for 2 min, followed by 30 cycles of denaturation at 94°C for 30 s, annealing at 50°C for 30 s, and elongation at 72°C for 1 min; with a final elongation step at 72°C for 5 min. CHIKV’s 5′UTR (5’untranslated region) and 3′UTR (3'-untranslated region) were amplified using SMARTer^®^ RACE 5′/3′ Kit (Takara Bio USA, Inc.). The PCR products were confirmed by agarose gel electrophoresis and sequenced by Sanger sequencing (Sangon Biotech, Shanghai). The full length of CHIKV genomic sequence was assembled on basis of 12 fragments, 5′UTR, and 3′UTR.

### Phylogenetic Analysis

The full length or nearly full length of CHIKV genomic sequences were downloaded from the GenBank database (https://www.ncbi.nlm.nih.gov/genbank/) and processed *via* the software SnapGene (GSL Biotech, USA). The nucleotide sequences were aligned using MUSCLE. The phylogenetic analysis based on nucleotide sequences of full genomes, structure proteins, and non-structure proteins was carried out using the maximum likelihood method with the Tamura-Nei model in MEGA7. The tree with the highest log likelihood (−34,152.40) is constructed.

### Analysis of Characteristic Mutations

Amino acid sequences of structural polyprotein (E1, E2, and E3) and non-structural polyprotein (nsP1, nsP2, nsP3, and nsP4) from 47 CHIKV strains, including four CHIKV strains of this study, were aligned with the prototypic CHIKV strain S27 *via* the software MEGA7. Furthermore, on basis of the prototypic CHIKV strain S27, SWISS-MODEL was used for comparative homology modeling of E1 and E2 of four strains identified in this study. Superimposed model was then visualized through PyMOL. The mutations identified in the study were highlighted as Stick format.

## Results

### Co-Infection of Chikungunya Virus with Dengue Virus in Febrile Patients

From September to November of 2019 during the epidemic dengue fever, we collected 697 serum samples from febrile dengue fever–like outpatients in Xishuangbanna Dai Autonomous Prefecture People’s Hospital, Yunnan, China. They were all local cases without any travel history. Multiplex one-step RT-PCR was performed to amplify gene fragments *via* specific primers for DENV (I–IV), CHIKV, and ZIKV. Sequences of PCR products were aligned with published viral sequences to confirm the specific pathogens. Among a total of 697 samples from febrile patients, 695 were positive for at least one of DENV, CHIKV, and ZIKV, and there were two samples negative for any of DENV, CHIKV, or ZIKV. Six hundred and ninety-three samples were DENV positive, accounting for 99.42% (693/697), among which DENV only was 87.09% (607/697), and coinfection of DENV and CHIKV was 12.33% (86/697). CHIKV only was 0.29% (2/697). Therefore, there were a total of 88 CHIKV-positive cases. Detailed results of PCR analysis were summarized in **Table 1**.

**Table 1:** Summary of differential identification of serum samples *via* RT-PCR.

Pathogen			Case number	Percentage (%)	Summary	
					Case	Percentage (%)
CHIKV			2	0.29	2	0.29
	I	CHIKV	4	0.57	86	12.33
	II		11	1.58		
	III		2	0.29		
	I + II		68	9.76		
DENV	I + II + III		1	0.14		
	I		14	2	607	87.09
	II		184	26.4		
	III		1	0.14		
	I + II		400	57.39		
	I + II + III		8	1.15		
Non-CHIKV/DENV/ZIKV			2	0.29	2	0.29
Total			697	100.00	697	100.00

RNA was extracted and purified from serum samples. One-step RT-PCR was performed via primers specific for four genotypes of DENV, CHIKV, and ZIKV, respectively. The gene fragments obtained from RT-PCR were sequenced, which was blasted with viral sequences of DENV, CHIKV, and ZIKV on the NCBI server.

**Figure 1:**
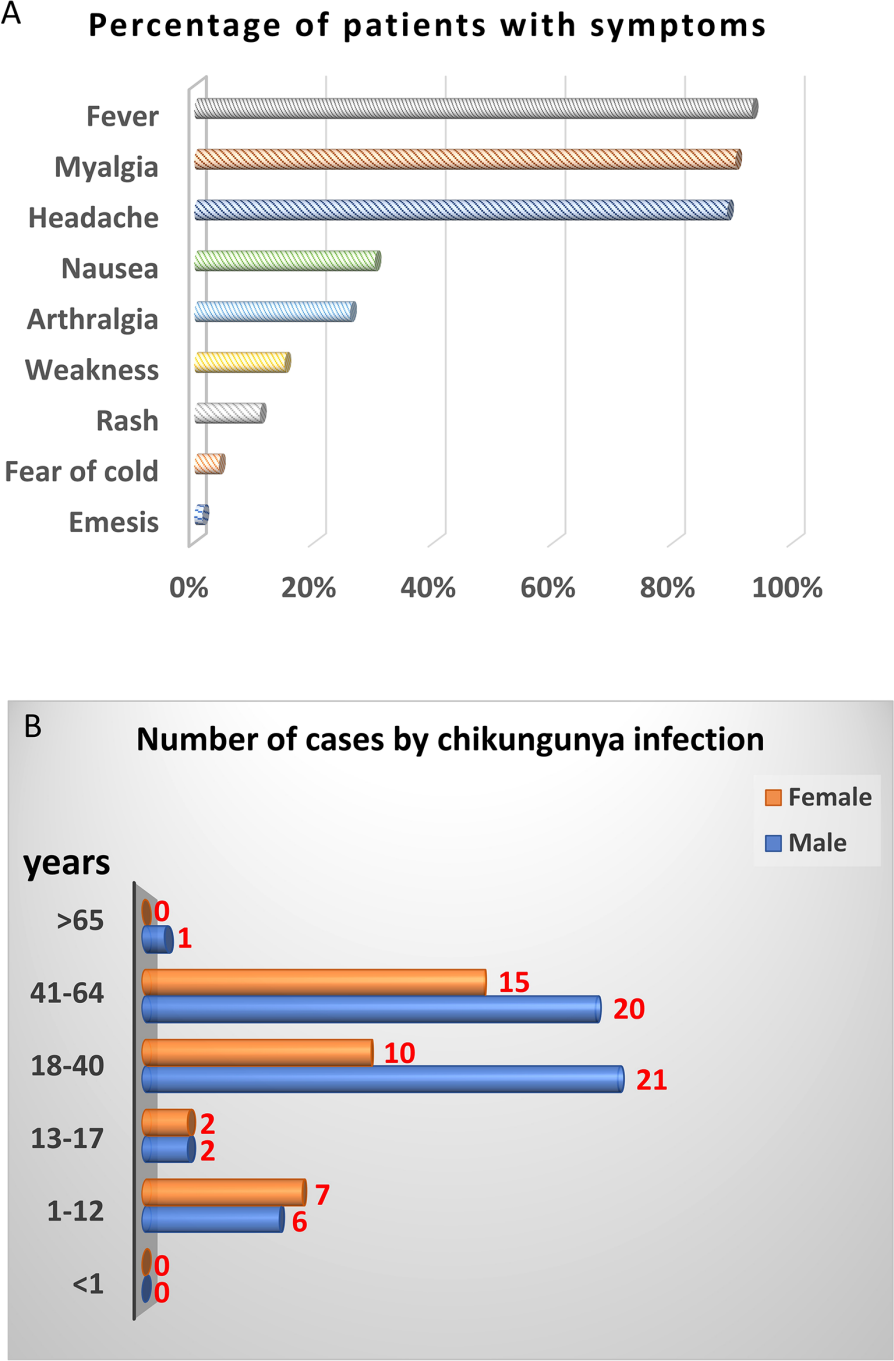
Demographic analysis of CHIKV-positive cases in this study. PCR and sequencing results showed that 88 out of 697 samples were CHIKV-positive. Fourteen CHIKV-positive patients were lack of symptom record. Therefore, only 74 CHIKV-positive patients with symptom record were chosen for demographic analysis. **(A)** Clinical symptoms. **(B)** Distribution of age and gender.

Next, we further analyzed demographics and symptoms of 74 CHIKV-positive cases except 14 CHIKV-positive cases lack of symptom record. The top three symptoms of CHIKV infection were fever, myalgia, and headache, accounting for 90%. About 30% of patients showed nausea, and 26.03% were arthralgia. The remaining patients showed weakness, rash, fear of cold, and emesis with 15.07%, 10.96%, 4.11%, and 1.37%, respectively (**Figure 1A**). In terms of gender and age, majority (66/88) of CHIKV-positive patients were at the age of 18–64 years, among which 31 cases were at the age of 18–40 years and 35 cases were at the age of 41–60 years. Male patients were more than female patients at the age of 18–64 years. There were 13 (seven females and six males) CHIKV-positive patients at the age of 1–12 years. Four (two females and two males) CHIKV-positive patients were at the age of 13–17 years. There was one patient with CHIKV older than 65 years (**Figure 1B**).

Furthermore, four CHIKV-positive serum samples, two from serum samples only with CHIKV and the other two from sera co-infected with DENV and CHIKV, were chosen for amplification of the whole genome *via* 12 pairs of primers, 5′RACE and 3′RACE (**Supplementary Table 1**). The full length of genomic sequences with 5′UTR and 3′UTR were eventually obtained from four CHIKV-positive samples and submitted to GenBank with accession numbers OK316990, OK316992, OK316993, and OK316995.

### Chikungunya Viruses Identified in Febrile Dengue Fever–Like Patients Belong to the Asian Lineage

**Figure 2:**
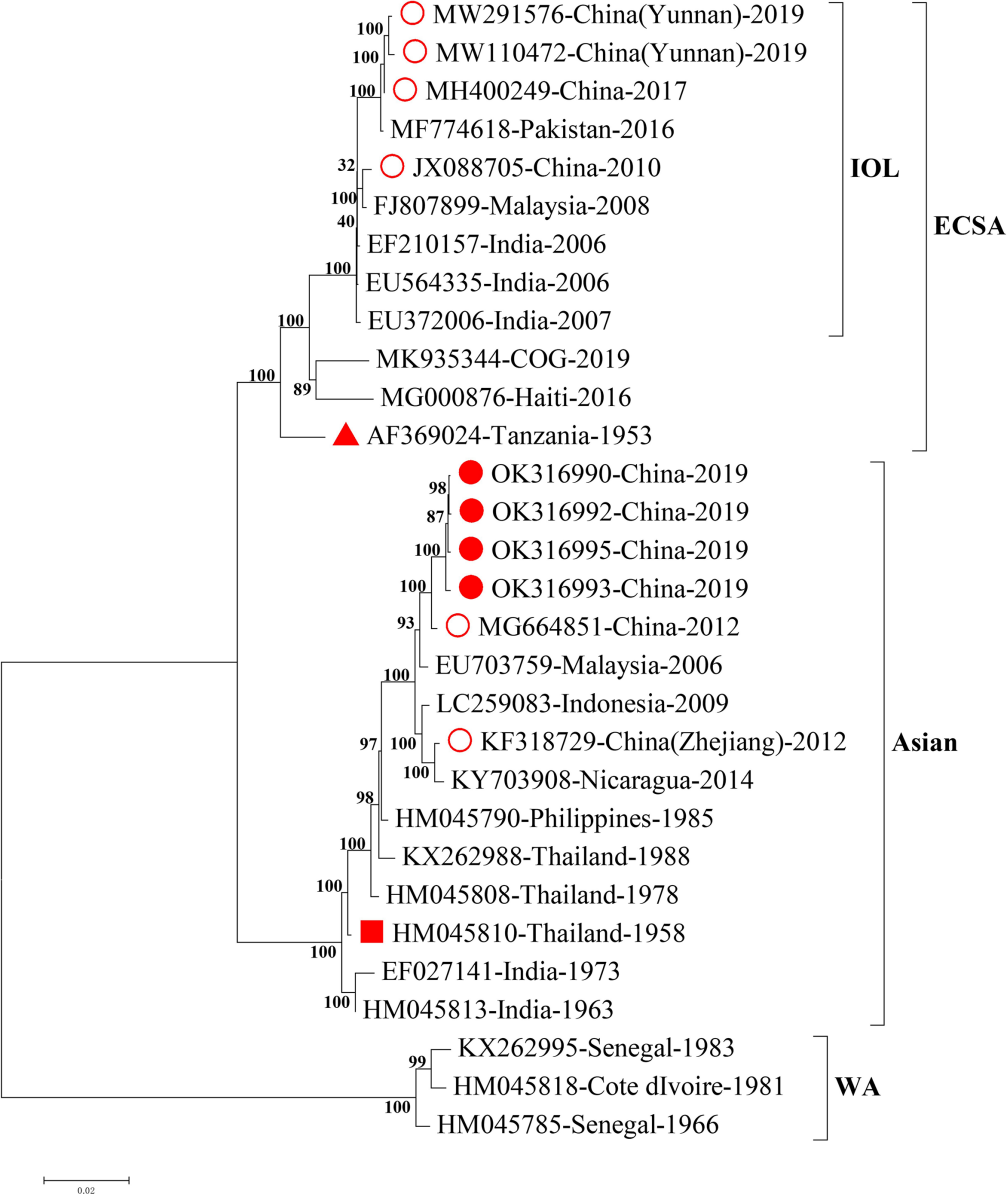
Phylogenetic analysis of the full length of CHIKV genomic sequences. Nearly complete sequences of representative CHIKV strains from each genotype were downloaded from NCBI GenBank database. Alignment and comparison were conducted to screen the genomic sequences with more than 59 bp at 5’UTR and more than 407 bp at 3’UTR for further phylogenetic analysis using MEGA7 maximum likelihood method with 500 bootstrap values. Representative strains of each genotype in the phylogenetic tree were named by accession number, country of origin, and year of isolation. The numbers on the branches of phylogenetic tree represent the posterior probability values. The red circles represent CHIKV strains isolated in China, the red dots for CHIKV strains isolated in this study, the red triangles for the earliest ECSA isolate S27, and the red squares for the earliest Asian isolate. The tree is drawn to scale, with branch lengths measured in the number of substitutions per site. CHIKV, chikungunya virus; WA, West African; ECSA, East/Central/South African; IOL, Indian Ocean Lineage.

To trace the potential origin of CHIKV identified in this study, we firstly compared the full length of CHIKV genomic sequences with those from the NCBI GenBank database. We found that the four CHIKV strains in this study shared 99.4% homology of nucleotide with the strain SZ1239 (accession number MG664851) in the Asian lineage and 91.2% of nucleotide homology with the strains in the sublineage Indian Ocean Lineage (IOL) of ECSA recently reported in Ruili, southwestern part of China (**Supplementary Table 2**) (Liu et al., 2021; Yin et al., 2021). The phylogenetic analysis of the full length of genomic sequences indicated that four CHIKV strains in this study were clustered to the Asian lineage with the CHIKV strain reported in 2012, China (**Figure 2**), distinct from the three strains documented in 2010, 2017, and 2019 in China that belong to the sublineage IOL of ESCA lineage. The gene fragments for structural proteins (C, E3, E1, and E2-6K) and non-structural proteins (nsP1, nsP2, nsP3, and nsP4) phylogenetically revealed four CHIKV strains in this study were also clustered in the Asian lineage (**Supplementary Figure 1**).

### Adaptive Mutations Observed in Chikungunya Viruses of This Study

Amino acid sequences of structure proteins and non-structure proteins of CHIKV strains in this study were aligned with those of the prototypic CHIKV strain S27 and others (**Table 2**; **Supplementary Table 3**). Notably, there were five of previously reported adaptive mutations also observed in the four CHIKV strains of this study, two in E1 protein (E1-A98T and E1-K211E), and three in E2 protein (E2-W64R, E2-I211T, and E2-K233E) (**Table 2**). In addition, there were eight mutations (E1-T65A, E1-V135I, E1-V291A, E1-A321V, E1-A329V, E2-N5R, E2-S27P, and E2-H123R/L) observed only in four CHIKV strains of this study (**Table 2**). In the non-structure proteins, four CHIKV strains of this study contained two adaptive mutations (nsP1-G230R and nsP3-R524G) (**Supplementary Table 3**).

**Table 2:** Amino acid mutations in the structural polyprotein of CHIKV.

Strain	Structure Protein																								
	E3	E2												E1											
	18	5	27	60	64	123	198	210	211	233	248	252	264	65	80	82	84	98	135	211	226	284	291	321	329
AF369024-Tanzania-1953	S	N	S	D	W	H	R	L	I	K	L	K	V	T	V	T	V	A	V	K	A	D	V	A	A
HM045812-Uganda-1982	.	.	.	G	.	.	.	.	.	.	.	.	.	.	.	.	.	.	.	.	.	.	.	.	.
KY575571-USA-2006	.	.	.	.	.	.	.	.	T	.	.	.	.	.	.	.	.	.	.	.	V	E	.	.	.
MK935344-COG-2019	.	.	.	.	.	.	.	.	.	.	.	.	.	.	.	.	.	.	.	.	V	.	.	.	.
FJ000069-India-2007	.	.	.	.	.	.	.	.	T	.	.	Q	.	.	.	.	.	.	.	.	V	E	.	.	.
FJ513628-Sri Lanka-2008	F	.	.	.	.	.	Q	.	T	.	.	.	.	.	.	.	.	.	.	.	V	E	.	.	.
FJ513657-Sri Lanka-2008	F	.	.	.	.	.	Q	.	T	.	.	.	.	.	.	.	.	.	.	.	V	E	.	.	.
FJ513675-Sri Lanka-2008	.	.	.	.	.	.	.	.	T	E	.	.	.	.	.	.	.	.	.	.	.	E	.	.	.
FJ807899-Malaysia-2008	.	.	.	.	.	.	.	.	T	.	.	Q	.	.	.	.	.	.	.	.	V	E	.	.	.
GU199352-China-2008	.	.	.	.	.	.	.	.	T	.	.	Q	.	.	.	.	.	.	.	.	V	E	.	.	.
KJ796845-India-2009	.	.	.	.	.	.	.	Q	T	.	.	.	.	.	.	.	.	.	.	.	V	E	.	.	.
MH124570-India-2010	.	.	.	.	.	.	.	.	T	.	.	.	A	.	.	.	.	.	.	E	.	E	.	.	.
KP003813-COG-2011	.	.	.	.	R	.	.	Q	T	.	.	.	.	.	.	.	.	.	.	.	V	.	.	.	.
MW581882-India-2013	.	.	.	.	.	.	.	.	T	.	.	.	A	.	.	.	.	.	.	E	.	E	.	.	.
MG925665.1-China(Henan)-2017	.	.	.	.	.	.	.	.	T	.	.	.	A	.	.	.	.	.	.	E	.	E	.	.	.
MH400249-China(Zhejiang)-2017	.	.	.	.	.	.	.	.	T	.	.	.	A	.	.	.	.	.	.	E	.	E	.	.	.
MT380159-Kenya-2018-Partial	.	.	.	.	.	.	.	.	T	.	.	.	A	.	A	I	D	.	.	E	-	-	-	-	-
MW110472-China(Yunnan)-2019	.	.	.	.	.	.	.	.	T	.	.	.	A	.	.	.	.	.	.	E	.	E	.	.	.
MW291576-China(Yunnan)-2019	.	.	.	.	.	.	.	.	T	.	.	.	A	.	.	.	.	.	.	E	.	E	.	.	.
**HM045785-Senegal-1966**	**.**	**.**	**.**	**.**	**.**	**.**	**.**	**.**	**T**	**.**	**.**	**.**	**.**	**.**	**.**	**.**	**.**	**.**	**.**	**.**	**.**	**.**	**.**	**T**	**.**
**HM045818-Cote d Ivoire-1981**	**.**	**.**	**.**	**.**	**.**	**.**	**.**	**.**	**T**	**.**	**.**	**.**	**.**	**.**	**.**	**.**	**.**	**.**	**.**	**.**	**.**	**.**	**.**	**.**	**.**
**KX262995-Senegal-1983**	**.**	**.**	**.**	**.**	**.**	**.**	**.**	**.**	**T**	**.**	**.**	**.**	**.**	**.**	**.**	**.**	**.**	**.**	**.**	**.**	**.**	**.**	**.**	**T**	**.**
HM045810-Thailand-1958	.	.	.	.	.	.	.	.	T	.	.	.	.	.	.	.	.	T	.	E	.	.	.	.	.
HM045813-India-1963	.	.	.	.	.	.	.	.	T	.	.	.	.	.	.	.	.	T	.	E	.	.	.	.	.
EF027141-India-1973	.	.	.	.	.	.	.	.	T	.	.	.	.	.	.	.	.	T	.	E	.	.	.	.	.
HM045808-Thailand-1978	.	.	.	.	.	.	.	.	T	.	.	.	.	.	.	.	.	T	.	E	.	.	.	.	.
HM045790-Philippines-1985	.	.	.	.	.	.	.	.	T	.	.	.	.	.	.	.	.	T	.	E	.	.	.	.	.
KX262988-Thailand-1988	.	.	.	.	.	.	.	.	T	.	.	.	.	.	.	.	.	T	.	E	.	.	.	.	.
EU703759-Malaysia-2006	.	H	.	.	.	.	.	.	T	.	S	.	.	.	.	.	.	T	.	E	.	.	.	.	.
MH670649-Malaysia-2009	.	H	.	.	.	.	.	.	T	.	S	.	.	.	.	.	.	T	.	E	.	.	.	.	.
LC259083-Indonesia-2009	.	H	.	.	.	.	.	.	T	.	F	.	.	.	.	.	.	T	.	E	.	.	.	.	.
KT308163-Philippines-2012	.	H	.	.	.	.	.	.	T	.	F	.	.	.	.	.	.	T	.	E	.	.	.	.	.
MG664851-China-2012	.	H	.	.	.	.	.	.	T	.	S	.	.	.	.	.	.	T	.	E	.	.	.	T	.
KF318729-China(Zhejiang)-2012	.	H	.	.	.	.	.	.	T	.	F	.	.	.	.	.	.	T	.	E	.	.	.	.	.
KX262991-Saint Martin-2013	.	H	.	.	.	.	.	.	T	.	F	.	.	.	.	.	.	T	.	E	.	.	.	.	.
KX262994-French-2014	.	H	.	.	.	.	.	.	T	.	F	.	.	.	.	.	.	T	.	E	.	.	.	.	.
KY575573-USA-2014	.	H	.	.	.	.	.	.	T	.	F	.	.	.	.	.	.	T	.	E	.	.	.	.	.
KY680411.1-USA-2014	.	H	.	.	.	.	.	.	T	.	F	.	.	.	.	.	.	T	.	E	.	.	.	.	.
KY680413.1-USA-2014	.	H	.	.	.	.	.	.	T	.	F	.	.	.	.	.	.	T	.	E	.	.	.	.	.
KY680414-USA-2014	.	H	.	.	.	.	.	.	T	.	F	.	.	.	.	.	.	T	.	E	.	.	.	.	.
KY703908-Nicaragua-2014	.	H	.	.	.	.	.	.	T	.	F	.	.	.	.	.	.	T	.	E	.	.	.	.	.
KY704000.1-Nicaragua-2015	.	H	.	.	.	.	.	.	T	.	F	.	.	.	.	.	.	T	.	E	.	.	.	.	.
OK655884.1-indonesia-2016	.	H	.	.	.	.	.	.	T	.	S	.	.	.	.	.	.	T	.	E	.	.	.	T	.
OK316990-China-2019	.	R	.	.	.	R	.	.	T	E	S	.	.	A	.	.	.	T	I	E	.	.	.	V	.
OK316992-China-2019	.	R	.	.	R	R	.	.	T	E	S	.	.	A	.	.	.	T	I	E	.	.	.	.	.
OK316993-China-2019	.	H	P	.	.	L	.	.	T	E	S	.	.	A	.	.	.	T	.	E	.	.	A	.	.
OK316995-China-2019	.	H	.	.	.	R	.	.	T	E	S	.	.	.	.	.	.	T	I	E	.	.	.	.	V

Amino acid sequences of E1, E2, and E3 from 47 CHIKV strains, including four CHIKV strains in this study, were aligned with the prototypic CHIKV strain S27. The reported adaptive mutations (highlighted in bold) and mutations only in four CHIKV strains in this study were listed in this table. The mutations with bold numbers also existed in CHIKV strains of this study.

To further predict the potential effects of mutations on the interaction between virus and host cell, three-dimensional structures of surface proteins E1 and E2 of four CHIKV strains in this study were modeled on basis of the prototypic strain S27 (accession number: AF369024) *via* the software PyMOL. Three mutations (E1-T65A, E1-V135I, and E1-V291A) in E1 protein of four CHIKV strains in this study did not make these residues protrude from the surface of E1 protein, but mutations of E1-A321V and E1-A329V resulted in obvious protrusion of amino acids (**Supplementary Figure 2A**). However, seven reported adaptive mutations in E1 protein led to their protruding from the surface of E1 protein, including E1-A98T and E1-K211E in CHIKV strains of this study. The mutation E2-N5R only observed in this study did not result in their protruding from the surface of E2 protein. The other two mutations (E2-S27P and E2-H123R/L) only in this study were revealed to be involved in interaction with the key residues Q63/Q64 and D68/R69 on MXRA8, one of important cellular receptor for CHIKV (Song et al., 2019), suggesting that MXRA8-binding property of CHIKV in this study may alter due to these two mutations. As expected, the nine reported adaptive mutations in E2 protein, including three mutations (E2-W64R, E2-I211T, and E2-K233E) observed in this study, resulted in their protruding from the surface of E2 protein (**Supplementary Figure 2B**).

## Discussion

Geographically, Yunnan province is located at the southwestern part of China and shares its border with several Southeast Asian countries (including Laos, Vietnam, and Myanmar) that are mostly affected by CHIKV. With the growth of tourism and trade with Southeast Asian countries, cases of imported CHIKV infection are constantly increasing, which may result in the re-emergence and autochthonous transmission of CHIKV to Yunnan. The present study highlights the urgent need for continuous molecular screening and epidemic surveillance for CHIKV and its vectors to prevent future outbreaks of CHIKV infection among the human population of Yunnan.

CHIKV was initially isolated from Tanzania (accession number AF369024, 1953), belonging to ECSA (Khan et al., 2002). The first CHIKV isolate in Asia can be traced to Thailand (accession number HM045810, 1958) (Volk et al., 2010). During 2018–2019, a number of imported and indigenous chikungunya cases were found in Yunnan Province, Southwest China (Xu et al., 2020; Liu et al., 2021; Yin et al., 2021). In addition molecular epidemiology revealed that CHIKV identified in this study was indigenous and belongs to Asian lineage, which is distinct from the reported CHIKV (ECSA lineage) in Ruili, the city next to Xishuangbanna, 2019 (Liu et al., 2021; Yin et al., 2021). However, there may be other lineages of CHIKV including ESCA in this outbreak since we sequenced and analyzed only four strains of CHIKV in this study. In fact, CHIKV RNA in the other 84 samples is not sufficient for further analysis. Therefore, we should incubate serum samples with susceptible cells to isolate and amplify CHIKV in the future to get more viral RNA. It is also very important that further studies should be conducted to trace the origin of CHIKV identified in this study.

Co-infection of DENV and CHIKV has been recently reported in several countries, which attracts great attentions due to its possibility of more severe diseases (Vogels et al., 2019). A multicenter study in Pakistan reveals the 12% of CHIKV co-infection in 590 DENV-positive patients at the average age of 28 (Raza et al., 2021). In India, a study in the population of 21- to 40-year-old patients shows 9.54% of DENV and CHIKV co-infection (Kaur et al., 2018). The results in these two reports are consistent with this study (**Table 1**). Unxpectedly, 35.7% of co-infection DENV and CHIKV has been reported in children under 18 years old (Castellanos et al., 2021). However, genotypes of DENV in CHIKV samples and the lineage of co-infected CHIKV have not been analyzed in these reported studies of co-infection. Thrombocytopenia and lymphopenia are frequently documented in dengue, chikungunya, or co-infected patients (Kaur et al., 2018). Patients with dengue show more severe thrombocytopenia and lymphopenia than patients with chikungunya (Tomashek et al., 2017). Coinfection of CHIKV may increase the severity of thrombocytopenia and lymphopenia in dengue patients (Singh et al., 2018). To further understand the mutual effects between DENV and CHIKV during the coinfection in this study, it is necessary to perform the routine laboratory test.

With the alignment of amino acid sequences, there were five of previously reported adaptive mutations also observed in the four CHIKV strains of this study, two in E1 protein (E1-A98T and E1-K211E), and three in E2 protein (E2-W64R, E2-I211T, and E2-K233E) (**Table 2**). E1-A98T mutation located in the fusion loop of the E1 protein (E1: 83–100 aa) occurs only in the Asian lineage and inhibits E1-A226V mutation (Zhang et al., 2018). The mutation E1-K211E significantly enhances the adaptability of CHIKV to *Aedes aegypti* in the context of E1-A226 (Agarwal et al., 2016). The residue E2-W64 is located at the tip of the spike protein (52–82 aa) and interacts with cellular receptors such as MXRA8 (Song et al., 2019). The mutation E2-W64R, also observed in one CHIKV isolate of this study, has been reported to show the decreased dissemination in *Ae. aegypti* but not *Ae. Albopictus* (Tsetsarkin et al., 2014). E2-I211T interacts with E1’s fusion ring and mediates virus entry, which is reported to significantly affect the infectivity of E1-A226V mutant in *Ae. albopictus* (Tsetsarkin et al., 2009). E2-K233E can directly enhance the infectivity of CHIKV in *Ae. albopictus*, mainly by alteration of viral infection and/or replication at the initial midgut (Berry et al., 2019). These reported adaptive mutations indicate that all of four CHIKV isolates in this study are potentially of high infectivity and adaption to both *Ae. aegypti* and *Ae. albopictus*, which probably increases the risk of arthropod-borne CHIKV infection in the population. In the non-structure proteins, four CHIKV strains of this study contained two adaptive mutations (nsP1-G230R and nsP3-R524G) (**Supplementary Table 3**). NsP1-G230R is reported to work together with nsP3-*524 to enhance the replication of CHIKV in *Ae. albopictus* (Mounce et al., 2017). The mutation nsP3-524R can attenuate arthritis and pathology in a mouse model, including reducing swelling and inflammation in the feet and ankles of mice, and inhibiting recruitment of pathological immune mediators, compared with the parent virus containing nSP3-*524 (Jones et al., 2017).

Through homology modeling, we hypothesize that mutations of E1-A321V and E1-A329V resulted in obvious protrusion of amino acids and MXRA8-binding property of CHIKV in this study may alter due to the two mutations (E2-S27P and E2-H123R/L). More experiments are necessary to test this hypothesis.

In summary, this study reports a DENV epidemic accompanied by CHIKV infection in Xishuangbanna, southwest part of China, in 2019. CHIKV from four identified cases in this study were characterized to be distinct from CHIKV previously reported in China. CHIKV strains in this study show the mutations potentially associated with enhanced infectivity for *Ae. aegypti* or *Ae. albopictus*. These findings provide important reference and guidance for the prevention and control of arboviruses such as DENV and CHIKV.

## Data Availability Statement

The datasets presented in this study can be found in online repositories. The names of the repository/repositories and accession number(s) can be found in the article/**Supplementary Material**.

## Ethics Statement

The studies involving human participants were reviewed and approved by Xishuangbanna Dai Autonomous Prefecture People’s Hospital, Yunnan, China. Written informed consent to participate in this study was provided by the participants’ legal guardian/next of kin. Written informed consent was obtained from the individual(s)’ and minor(s)’ legal guardian/next of kin, for the publication of any potentially identifiable images or data included in this article.

## Author Contributions

HL, XP, and QS contributed to the design of the study. HL and MZ were involved in writing this manuscript. MZ, CS, JL, and NL were involved in data acquisition and analysis. JZ, TL, DM, and DL contributed to collection of samples and clinical information. All authors contributed to the article and approved the submitted version.

## Funding

This work was supported by funding from Yunnan Key R&D project (202103AQ100001), Chinese Central Government for Basic Scientific Research Operations in Commonweal Research Institutes (2021-PT310-007), CAMS Innovation Fund for Medical Sciences (CIFMS, 2021-I2M-1-043), Yunnan Provincial Key Laboratory of Vector-borne Diseases Control and Research (2015DG037), and Innovation Team Project of Yunnan Science and Technology Department (202105AE160020).

## Conflict of Interest

The authors declare that the research was conducted in the absence of any commercial or financial relationships that could be construed as a potential conflict of interest.

## Publisher’s Note

All claims expressed in this article are solely those of the authors and do not necessarily represent those of their affiliated organizations, or those of the publisher, the editors and the reviewers. Any product that may be evaluated in this article, or claim that may be made by its manufacturer, is not guaranteed or endorsed by the publisher.
