# Supplementary material for: Genetic Characterization of Chikungunya Virus Among Febrile Dengue Fever–Like Patients in Xishuangbanna, Southwestern Part of China
Source: Front Cell Infect Microbiol. 2022 Jun 27;12:914289. doi: 10.3389/fcimb.2022.914289 (PMC9271616; doi:10.3389/fcimb.2022.914289)
Supplement: Supplementary file 1 [file Table_1.docx]

**
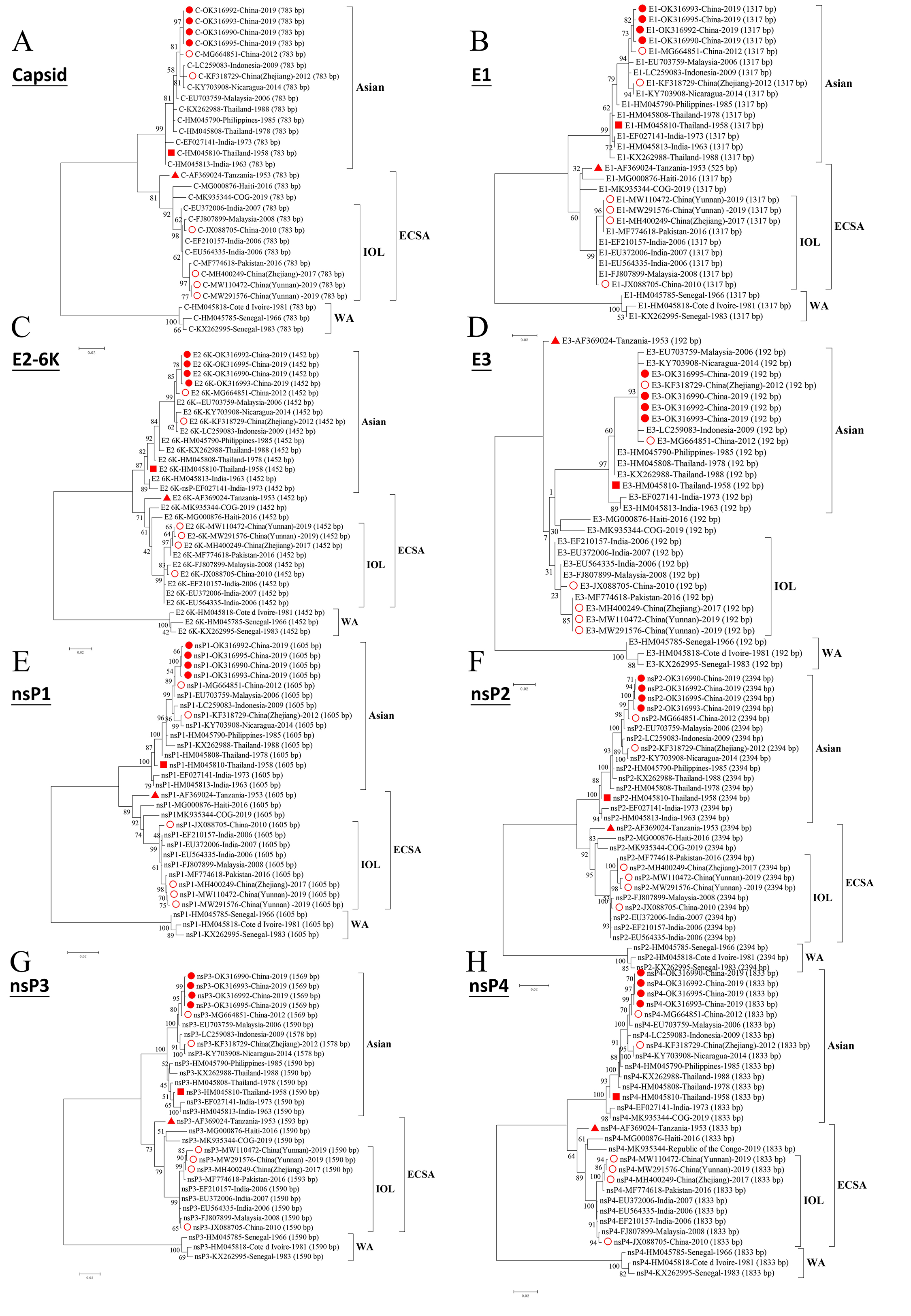
**

**Supplementary Figure 1.** Phylogenetic analysis of nucleotide sequences of CHIKV gene fragments. Nearly complete sequences of representative CHIKV strains from each genotype were downloaded from NCBI Genbank database. The coding sequences for structure proteins (Capsid from 1 to 261aa, E3: from 262 to 325aa, E2-6k: from 326 to 809aa, E1: from 810 to 1248aa.) and non-structural proteins (nsP1 from 1 to 535aa, nsP2 from 536 to 1333aa, nsP3 from 1334 to 1856aa, nsP4 from 1857 to 2467aa.) were extracted from the genomic sequences. Representative strains of each genotype were named by accession number, country of origin, and year of isolation. The numbers on the branches represented the posterior probability values. The red circles represent CHIKV strains isolated in China, the red dots for CHIKV strains isolated in this study, the red triangles for the earliest ECSA isolate S27, and the red squares for the earliest Asian isolate. Scale bar indicates nucleotide variation. CHIKV, chikungunya virus; WA, West African; ECSA, East/Central/South African; IOL, Indian Ocean Lineage.

**Supplementary Figure 2.** Tridimensional localization of amino acid exchanges in the structural proteins E1 and E2 of CHIKV. On basis of the prototypic CHIKV strain S27, Swiss-Model was used for comparative homology modeling of E1 (A) and E2 (B) of 4 strains identified in this study. Superimposed model was then visualized through Pymol. The selected mutations identified in the study were highlighted as Stick format. Letters in red color are the sites of adaptive amino acid mutations reported. The red in black boxes represent adaptive amino acid exchanges in CHIKV strains of this study. The red with underlines means no amino acid exchange aligned with those in the prototypic strain S27. Letters in yellow color are for unique exchange of amino acids only in this study.

**Supplementary Table 1.**

List of primers for amplification of CHIKV genomic fragments via RT-PCR

| Primers | Sequences | |  |
| --- | --- | --- | --- |
| 5’-GSP1-1 | | GATTACGCCAAGCTTCCGACATCATCCTCCTTGCTGGCGCAC | |
| 5’-GSP1-2 | | GATTACGCCAAGCTTCGCCTGGTGATATAGCGACGTGGGTGC | |
| 3’-GSP2-1 | | GATTACGCCAAGCTTCACGGGAGGTGTGGGACTGGTTGTCGC | |
| 3’-CDS Primer A | | AAGCAGTGGTATCAACGCAGAGTACTTTTTTTTTTTTTTTTTTTTT  TTTTTTTTTVN | |
| CHIKV-Q-1.0F | | ATAGCTGCGTGAGACACTCG | |
| CHIKV-Q-1R | | CTCATCTTCCATGTCCTTCC | |
| CHIKV-Q-2F | | TTGATGTGCAAGACTACCGAC | |
| CHIKV-Q-2R | | ATGGCTGACTTACCAGATCCT | |
| CHIKV-Q-3F | | AACACGAGTACGTCTACGAC | |
| CHIKV-Q-3R | | CCGCGTAATACACAGATACC | |
| CHIKV-Q-4F | | TCTGCAGTCACCAAGTGACC | |
| CHIKV-Q-4R | | TGCGACTTCTCGATAGGCAG | |
| CHIKV-Q-5F | | TGGACATCGCGAAGAACGAT | |
| CHIKV-Q-5R | | TCACATACCACGGTCAGGTT | |
| CHIKV-Q-6F | | TACAGATCTTCCCAGGAGTC | |
| CHIKV-Q-6R | | CGTTGAATACTGCTGAGTCC | |
| CHIKV-Q-7F | | CACCGATGAGTATGATGCAT | |
| CHIKV-Q-7R | | TTCTGTCTTCGTCTTGTTCG | |
| CHIKV-Q-8F | | TCCGATGAATTGATGGCAGC | |
| CHIKV-Q-8R | | TGTACGGGCTCCTTCATTAG | |
| CHIKV-Q-9F | | TAAGTACGACCTTGAATGCG | |
| CHIKV-Q-9R | | GCATGGCATTGATCAACCTT | |
| CHIKV-Q-10F | | CCGAGGAGATAGAGGTACAT | |
| CHIKV-Q-10R | | CAGCTGTAATCAGGTAGGCT | |
| CHIKV-Q-11F | | AGACTCTAGTCAACAGACCG | |
| CHIKV-Q-11R | | GTTGTCAAGTTAGTGCCTGC | |
| CHIKV-Q-12F | | CCATCATTAAATATGCAGCC | |
| CHIKV-Q-12R | | CAAAATAACATCTCCTAC | |

**Supplementary Table 2.**

Homology analysis of the whole genome sequences of 30 CHIKV strains

Note: The sequences in this table were also used for the phylogenetic analysis in Figure 2 and Figure S2.

**Supplementary Table 3.**

Amino acid mutations in non-structural polyprotein of CHIKV

| **Strain** | **non-structure proteins** | | | | | | | | | | | | | | | | |
| --- | --- | --- | --- | --- | --- | --- | --- | --- | --- | --- | --- | --- | --- | --- | --- | --- | --- |
|  | nsP1 | | | | | nsP2 | | | | | nsP3 | | | | nsP4 | | |
|  | 27 | 187 | **230** | 326 | 398 | 202 | 452 | 580 | 747 | 790 | 38 | 117 | 494 | **524** | 49 | 290 | 494 |
| AF369024-Tanzania-1953 | V | A | G | V | C | R | V | N | S | V | Y | G | L | R | L | M | I |
| HM045812-Uganda-1982 | . | . | . | . | . | . | . | . | . | . | . | . | . | X | . | . | . |
| KY575571-USA-2006 | . | . | . | . | . | . | . | . | . | . | . | . | . | . | . | . | . |
| MK935344-COG-2019 | . | . | . | . | . | . | . | . | . | . | . | . | . | - | . | . | . |
| FJ000069-India-2007 | . | . | . | . | . | . | . | . | . | . | . | . | . | X | . | . | . |
| FJ513628-Sri Lanka-2008 | . | . | . | . | . | . | . | . | . | . | H | . | . | X | . | . | . |
| FJ513657-Sri Lanka-2008 | . | . | . | . | . | . | . | . | . | . | H | . | . | X | . | . | . |
| FJ513675-Sri Lanka-2008 | . | . | R | . | . | . | . | . | . | . | . | . | . | X | . | . | . |
| FJ807899-Malaysia-2008 | . | . | . | . | . | . | . | . | . | . | . | . | . | - | . | . | . |
| GU199352-China-2008 | . | . | . | . | . | . | . | . | . | . | . | . | . | - | . | . | . |
| KJ796845-India-2009 | . | . | . | . | . | . | . | . | . | . | . | . | . | - | . | . | . |
| MH124570-India-2010 | . | . | R | . | . | . | . | . | . | . | . | . | . | . | . | . | . |
| KP003813-COG-2011 | . | . | . | . | . | . | . | . | . | . | . | . | . | X | . | . | . |
| MW581882-India-2013 | . | . | R | . | . | . | S | . | . | . | . | . | . | . | . | . | . |
| MG925665.1-China henan-2017 | . | . | . | . | . | . | . | . | . | . | . | . | . | X | . | . | . |
| MH400249-China-2017 | . | . | . | . | . | . | . | . | . | . | . | . | . | - | . | . | . |
| MT380159-Kenya-2018-Partial | . | . | . | . | . | . | . | . | . | . | . | . | . | X | . | . | . |
| MW110472-China(Yunnan)-2019 | . | . | . | . | . | . | . | . | . | . | . | . | . | - | . | . | . |
| MW291576-China(Yunnan)-2019 | . | . | . | . | . | . | . | . | . | . | . | . | . | - | . | . | . |
| HM045785-Senegal-1966 | . | . | . | . | . | . | . | . | . | . | . | . | . | - | . | . | . |
| HM045818-Cote d Ivoire-1981 | . | . | . | . | . | . | . | . | . | . | . | . | . | - | . | . | . |
| KX262995-Senegal-1983 | . | . | . | . | . | . | . | . | . | . | . | . | . | - | . | . | . |
| HM045810-Thailand-1958 | . | . | . | . | . | . | . | . | . | . | . | . | . | - | . | . | . |
| HM045813-India-1963 | . | . | . | . | . | . | . | . | . | . | . | . | . | - | . | . | . |
| EF027141-India-1973 | . | . | . | . | . | . | . | . | . | . | . | . | . | - | . | . | . |
| HM045808-Thailand-1978 | . | . | . | . | . | . | . | . | . | . | . | . | . | - | . | . | . |
| HM045790-Philippines-1985 | . | . | . | . | . | . | . | . | . | . | . | . | . | - | . | . | . |
| KX262988-Thailand-1988 | . | . | . | M | . | . | . | . | . | . | . | . | . | - | . | . | . |
| EU703759-Malaysia-2006 | . | . | . | . | . | . | . | . | . | . | . | . | . | - | . | . | . |
| MH670649-Malaysia-2009 | . | . | . | . | . | . | . | . | . | . | . | . | . | G | . | . | . |
| LC259083-Indonesia-2009 | . | . | . | . | . | . | . | . | . | . | . | . | . | - | . | . | . |
| KT308163-Philippines-2012 | . | . | . | . | . | . | . | . | . | . | . | . | . | - | . | . | . |
| MG664851-China-2012 | . | . | . | . | . | . | . | . | . | . | . | . | . | G | . | . | . |
| KF318729-China(Zhejiang)-2012 | . | . | . | . | . | . | . | . | . | . | . | . | . | - | . | . | . |
| KX262991-Saint Martin-2013 | . | . | . | . | . | . | . | . | . | . | . | . | . | . | . | . | . |
| KX262994-French-2014 | . | . | . | . | . | . | . | . | . | . | . | . | . | - | . | . | . |
| KY575573-USA-2014 | . | . | . | . | . | . | . | . | . | . | . | . | . | . | . | . | . |
| KY680411.1-USA-2014 | . | . | . | . | . | . | . | . | . | . | . | . | . | . | . | . | . |
| KY680413.1-USA-2014 | . | . | . | . | . | . | . | . | . | . | . | . | . | . | . | . | . |
| KY680414-USA-2014 | . | . | . | . | . | . | . | . | . | . | . | . | . | - | . | . | . |
| KY703908-Nicaragua-2014 | . | . | . | . | . | . | . | . | . | . | . | . | . | - | . | . | . |
| KY704000.1-Nicaragua-2015 | . | . | . | . | . | . | . | . | . | . | . | . | . | . | . | . | . |
| OK655884.1-indonesia-2016 | . | . | . | . | . | . | . | . | . | . | . | . | . | X | . | . | . |
| OK316990-China-2019 | A | . | R | . | R | T | . | Y | . | . | . | R | . | G | H | L | T |
| OK316992-China-2019 | . | . | R | . | R | T | . | Y | . | . | . | R | P | G | H | L | T |
| OK316993-China-2019 | . | T | R | . | . | . | . | Y | A | A | . | R | . | G | H | L | . |
| OK316995-China-2019 | . | . | R | . | R | T | A | Y | . | . | . | R | . | G | H | L | T |

Note: Amino acid sequences of nonstructural proteins from more than 80 CHIKV strains, including four CHIKV strains in this study, were aligned with the prototypic CHIKV strain S27. The reported adaptive mutations (highlighted in blue background) and mutations only in four CHIKV strains in this study were listed in this table. The mutations with bold numbers also existed in CHIKV strains of this study.
