# Supplementary material for: Genetic Characterization of Chikungunya Virus Among Febrile Dengue Fever–Like Patients in Xishuangbanna, Southwestern Part of China
Source: Front Cell Infect Microbiol. 2022 Jun 27;12:914289. doi: 10.3389/fcimb.2022.914289 (PMC9271616; doi:10.3389/fcimb.2022.914289)
Supplement: Supplementary file 2 [file Table_2.docx]

**Supplementary Table 2.**

Homology analysis of the whole genome sequences of 30 CHIKV strains

|  |  | **Percent identity** | | | | | | | | | | | | | | | | | | | | | | | | | | | | | |  |
| --- | --- | --- | --- | --- | --- | --- | --- | --- | --- | --- | --- | --- | --- | --- | --- | --- | --- | --- | --- | --- | --- | --- | --- | --- | --- | --- | --- | --- | --- | --- | --- | --- |
|  |  | **1** | **2** | **3** | **4** | **5** | **6** | **7** | **8** | **9** | **10** | **11** | **12** | **13** | **14** | **15** | **16** | **17** | **18** | **19** | **20** | **21** | **22** | **23** | **24** | **25** | **26** | **27** | **28** | **29** | **30** |  |
| **Divergence** | **1** | *** | 94.8 | 97.1 | 97.1 | 97.2 | 92.7 | 97.1 | 85.1 | 92.7 | 93.4 | 93.8 | 93.7 | 84.9 | 97 | 92.5 | 94.1 | 84.8 | 92 | 92.7 | 96.7 | 97 | 92.3 | 96.7 | 97 | 96.5 | 96.6 | 91.9 | 91.9 | 91.9 | 91.9 | AF369024-Tanzania-1953 |
|  | **2** | 5.1 | *** | 94.2 | 94.2 | 94.3 | 97.4 | 94.1 | 84.2 | 97.5 | 98.4 | 99 | 99.5 | 84.1 | 94 | 96.9 | 97.9 | 83.8 | 95.6 | 97.1 | 94.1 | 94.2 | 96.6 | 94 | 94.2 | 94 | 93.9 | 96.3 | 96.3 | 96.4 | 96.4 | EF027141-India-1973 |
|  | **3** | 2.9 | 5.8 | *** | 99.9 | 99.9 | 93.6 | 99.7 | 84.9 | 93.8 | 94.1 | 94.6 | 94.5 | 84.8 | 99.6 | 93.4 | 93.9 | 84.7 | 91.9 | 93.5 | 99.2 | 97.5 | 93.2 | 99.3 | 97.6 | 99 | 99.1 | 92.8 | 92.8 | 92.8 | 92.9 | EF210157-India-2006 |
|  | **4** | 2.9 | 5.8 | 0.1 | *** | 99.8 | 93.6 | 99.7 | 84.9 | 93.8 | 94.1 | 94.5 | 94.5 | 84.7 | 99.5 | 93.4 | 94 | 84.7 | 91.9 | 93.5 | 99.2 | 97.4 | 93.2 | 99.2 | 97.6 | 99 | 99.1 | 92.8 | 92.8 | 92.8 | 92.9 | EU372006-India-2007 |
|  | **5** | 2.8 | 5.7 | 0.1 | 0.2 | *** | 92.3 | 99.8 | 85 | 92.4 | 92.9 | 93.2 | 93.2 | 84.8 | 99.6 | 92.1 | 93.6 | 84.7 | 91.5 | 92.3 | 99.3 | 97.6 | 91.9 | 99.3 | 97.7 | 99.1 | 99.2 | 91.5 | 91.5 | 91.5 | 91.5 | EU564335-India-2006 |
|  | **6** | 6 | 2.6 | 6.6 | 6.6 | 6.5 | *** | 92.2 | 83.6 | 98 | 98.4 | 98 | 97.7 | 83.5 | 92.1 | 99 | 98.5 | 83.3 | 97.4 | 99.1 | 92 | 92.1 | 99 | 93.5 | 93.5 | 91.9 | 92 | 98.6 | 98.6 | 98.6 | 98.7 | EU703759-Malaysia-2006 |
|  | **7** | 3 | 5.9 | 0.3 | 0.3 | 0.2 | 6.7 | *** | 84.9 | 92.3 | 92.8 | 93.1 | 93.1 | 84.7 | 99.7 | 92 | 93.5 | 84.6 | 91.4 | 92.1 | 99.1 | 97.4 | 91.7 | 99.2 | 97.5 | 98.9 | 99 | 91.4 | 91.4 | 91.4 | 91.4 | FJ807899-Malaysia-2008 |
|  | **8** | 16.5 | 17.6 | 16.8 | 16.8 | 16.7 | 18.3 | 16.8 | *** | 83.8 | 84.1 | 84.3 | 84.4 | 99 | 84.9 | 83.5 | 84 | 98 | 82.6 | 83.5 | 85 | 85.1 | 83.3 | 84.8 | 84.9 | 84.8 | 84.8 | 83.1 | 83.1 | 83.1 | 83.1 | HM045785-Senegal-1966 |
|  | **9** | 5.4 | 1.9 | 5.9 | 5.9 | 5.9 | 1.2 | 6 | 17.9 | *** | 98.7 | 98.2 | 97.9 | 83.7 | 92.2 | 97.6 | 98.6 | 83.4 | 96.1 | 97.7 | 92.2 | 92.2 | 97.8 | 93.6 | 93.6 | 92.1 | 92.1 | 97.5 | 97.5 | 97.5 | 97.5 | HM045790-Philippines-1985 |
|  | **10** | 5.3 | 1.6 | 5.9 | 5.9 | 5.8 | 1.4 | 6 | 17.8 | 0.6 | *** | 99.1 | 98.8 | 84 | 92.7 | 98 | 99.2 | 83.7 | 96.6 | 98.2 | 92.8 | 92.8 | 97.8 | 93.9 | 94 | 92.7 | 92.6 | 97.4 | 97.4 | 97.5 | 97.5 | HM045808-Thailand-1978 |
|  | **11** | 4.7 | 1 | 5.3 | 5.4 | 5.3 | 1.9 | 5.4 | 17.4 | 1.1 | 0.8 | *** | 99.5 | 84.2 | 93 | 97.6 | 98.6 | 83.9 | 96.2 | 97.7 | 93.1 | 93.1 | 97.3 | 94.3 | 94.5 | 93 | 92.9 | 97 | 97 | 97 | 97 | HM045810-Thailand-1958 |
|  | **12** | 4.8 | 0.5 | 5.5 | 5.5 | 5.4 | 2.2 | 5.5 | 17.4 | 1.4 | 1.2 | 0.5 | *** | 84.3 | 93 | 97.3 | 98.3 | 83.9 | 95.9 | 97.3 | 93.1 | 93.1 | 97 | 94.3 | 94.4 | 93 | 92.9 | 96.7 | 96.7 | 96.7 | 96.7 | HM045813-India-1963 |
|  | **13** | 16.8 | 17.8 | 17 | 17.1 | 17 | 18.4 | 17.1 | 1 | 18 | 17.9 | 17.6 | 17.6 | *** | 84.7 | 83.4 | 83.9 | 98.4 | 82.6 | 83.5 | 84.8 | 84.9 | 83.2 | 84.7 | 84.8 | 84.6 | 84.6 | 83 | 83 | 83 | 83 | HM045818-Cote d Ivoire-1981 |
|  | **14** | 3.1 | 6 | 0.4 | 0.5 | 0.4 | 6.8 | 0.3 | 16.9 | 6.1 | 6.1 | 5.6 | 5.6 | 17.2 | *** | 91.9 | 93.4 | 84.6 | 91.3 | 92.1 | 99 | 97.3 | 91.7 | 99.1 | 97.4 | 98.8 | 98.9 | 91.3 | 91.3 | 91.3 | 91.3 | JX088705-China-2010 |
|  | **15** | 6.2 | 3 | 6.7 | 6.7 | 6.6 | 0.9 | 6.8 | 18.3 | 1.5 | 1.8 | 2.2 | 2.5 | 18.4 | 6.9 | *** | 98.2 | 83.2 | 98.2 | 99.3 | 91.7 | 91.8 | 98.6 | 93.2 | 93.3 | 91.7 | 91.8 | 98.3 | 98.3 | 98.3 | 98.3 | KF318729-China(Zhejiang)-2012 |
|  | **16** | 5.5 | 2.1 | 6.1 | 6.1 | 6.1 | 1.5 | 6.2 | 17.9 | 0.6 | 0.8 | 1.2 | 1.6 | 18 | 6.3 | 1.8 | *** | 83.7 | 96.7 | 98.2 | 93.4 | 93.5 | 97.9 | 93.7 | 93.8 | 93.3 | 93.3 | 97.5 | 97.5 | 97.5 | 97.5 | KX262988-Thailand-1988 |
|  | **17** | 16.8 | 18 | 17.1 | 17.2 | 17.1 | 18.6 | 17.2 | 1.1 | 18.2 | 18.1 | 17.8 | 17.8 | 0.8 | 17.3 | 18.6 | 18.2 | *** | 82.4 | 83.3 | 84.6 | 84.8 | 83 | 84.5 | 84.6 | 84.5 | 84.5 | 82.8 | 82.8 | 82.8 | 82.8 | KX262995-Senegal-1983 |
|  | **18** | 6.2 | 2.9 | 6.7 | 6.7 | 6.7 | 1 | 6.8 | 18.3 | 1.6 | 1.8 | 2.2 | 2.5 | 18.4 | 6.9 | 0.3 | 1.9 | 18.6 | *** | 97.7 | 91.3 | 91.3 | 97.1 | 91.7 | 91.7 | 91.2 | 91.2 | 96.7 | 96.7 | 96.7 | 96.7 | KY703908-Nicaragua-2014 |
|  | **19** | 6 | 2.8 | 6.6 | 6.6 | 6.5 | 0.7 | 6.7 | 18.3 | 1.3 | 1.6 | 2 | 2.4 | 18.4 | 6.7 | 0.6 | 1.6 | 18.6 | 0.7 | *** | 91.9 | 92.1 | 98.8 | 93.3 | 93.4 | 91.9 | 91.9 | 98.4 | 98.4 | 98.4 | 98.4 | LC259083-Indonesia-2009 |
|  | **20** | 3.3 | 5.9 | 0.7 | 0.7 | 0.6 | 6.8 | 0.8 | 16.8 | 6.1 | 6.1 | 5.6 | 5.6 | 17.1 | 0.9 | 6.9 | 6.4 | 17.3 | 7 | 6.8 | *** | 97 | 91.6 | 99.8 | 97.1 | 99.6 | 99.6 | 91.2 | 91.2 | 91.2 | 91.2 | MF774618-Pakistan-2016 |
|  | **21** | 3.1 | 5.8 | 2.6 | 2.7 | 2.6 | 6.8 | 2.8 | 16.5 | 6.1 | 6 | 5.5 | 5.5 | 16.8 | 2.9 | 6.9 | 6.3 | 16.9 | 6.9 | 6.7 | 3.1 | *** | 91.7 | 97 | 97.5 | 96.8 | 96.9 | 91.3 | 91.3 | 91.3 | 91.3 | MG000876-Haiti-2016 |
|  | **22** | 6.2 | 3 | 6.7 | 6.7 | 6.7 | 0.6 | 6.8 | 18.4 | 1.4 | 1.7 | 2.1 | 2.5 | 18.5 | 6.9 | 1.1 | 1.7 | 18.7 | 1.2 | 0.9 | 6.9 | 6.9 | *** | 93 | 93 | 91.5 | 91.6 | 99.4 | 99.3 | 99.4 | 99.4 | MG664851-China-2012 |
|  | **23** | 3.4 | 6 | 0.7 | 0.8 | 0.6 | 6.7 | 0.8 | 16.9 | 6.1 | 6.1 | 5.6 | 5.7 | 17.2 | 0.9 | 6.9 | 6.4 | 17.3 | 7 | 6.8 | 0.1 | 3.1 | 6.9 | *** | 97.2 | 99.7 | 99.8 | 92.7 | 92.6 | 92.7 | 92.7 | MH400249-China-2017 |
|  | **24** | 3 | 5.8 | 2.5 | 2.5 | 2.4 | 6.8 | 2.6 | 16.9 | 6.1 | 6 | 5.4 | 5.5 | 17.1 | 2.7 | 6.9 | 6.3 | 17.2 | 7 | 6.7 | 3 | 2.6 | 6.9 | 3 | *** | 96.9 | 97.1 | 92.7 | 92.7 | 92.7 | 92.7 | MK935344-COG-2019 |
|  | **25** | 3.5 | 6.1 | 0.9 | 1 | 0.9 | 6.9 | 1.1 | 17.1 | 6.2 | 6.2 | 5.7 | 5.8 | 17.3 | 1.2 | 7 | 6.5 | 17.4 | 7.1 | 6.9 | 0.4 | 3.3 | 7 | 0.2 | 3.2 | *** | 99.7 | 91.1 | 91.1 | 91.1 | 91.2 | MW110472-China (Yunnan)-2019 |
|  | **26** | 3.5 | 6.1 | 0.9 | 0.9 | 0.8 | 6.8 | 1 | 17 | 6.2 | 6.2 | 5.7 | 5.8 | 17.3 | 1.1 | 7 | 6.4 | 17.4 | 7.1 | 6.9 | 0.3 | 3.3 | 7 | 0.2 | 3.1 | 0.2 | *** | 91.2 | 91.2 | 91.2 | 91.2 | MW291576-China (Yunnan)-2019 |
|  | **27** | 6.6 | 3.2 | 7.1 | 7.1 | 7 | 0.9 | 7.2 | 18.6 | 1.8 | 2 | 2.4 | 2.8 | 18.7 | 7.3 | 1.4 | 2 | 18.9 | 1.5 | 1.2 | 7.3 | 7.3 | 0.6 | 7.3 | 7.3 | 7.4 | 7.4 | *** | 99.9 | 99.8 | 99.9 | OK316990-China-2019 |
|  | **28** | 6.6 | 3.2 | 7.1 | 7.1 | 7.1 | 1 | 7.2 | 18.6 | 1.8 | 2 | 2.4 | 2.8 | 18.7 | 7.3 | 1.4 | 2.1 | 18.9 | 1.5 | 1.2 | 7.3 | 7.3 | 0.6 | 7.3 | 7.3 | 7.4 | 7.4 | 0.1 | *** | 99.8 | 99.9 | OK316992-China-2019 |
|  | **29** | 6.5 | 3.2 | 7.1 | 7.1 | 7 | 0.9 | 7.2 | 18.6 | 1.8 | 2 | 2.4 | 2.8 | 18.7 | 7.3 | 1.4 | 2 | 18.9 | 1.5 | 1.2 | 7.3 | 7.3 | 0.6 | 7.3 | 7.3 | 7.4 | 7.4 | 0.2 | 0.2 | *** | 99.8 | OK316993-China-2019 |
|  | **30** | 6.5 | 3.2 | 7.1 | 7 | 7 | 0.9 | 7.1 | 18.6 | 1.7 | 2 | 2.4 | 2.7 | 18.7 | 7.2 | 1.3 | 2 | 18.9 | 1.4 | 1.1 | 7.2 | 7.2 | 0.6 | 7.2 | 7.3 | 7.3 | 7.3 | 0.1 | 0.1 | 0.2 | *** | OK316995-China-2019 |

Note: The sequences in this table were also used for the phylogenetic analysis in Figure 2 and Supplementary Figure 1.
